# Specific nutrient combination effects on tax, NF- κB and MMP-9 in human T-cell lymphotropic virus -1 positive malignant T-lymphocytes

**DOI:** 10.1186/1471-2407-15-S1-S2

**Published:** 2015-01-15

**Authors:** Steve Harakeh, Rania Azar, Esam Azhar, Ghazi A Damanhouri, Mourad Assidi, Muhammad Abu-Elmagd, Mohammed H Alqahtani, Taha Kumosani, Aleksandra Niedzwiecki, Mathias Rath, Ahmed Al-Hejin, Elie Barbour, Mona Diab-Assaf

**Affiliations:** 1 Special infectious agents unit , King Fahd Medical Research Center, King Abdulaziz University, P.O. Box 80216 Jeddah 21589, Kingdom of Saudi Arabia; 2 Molecular Tumorigenesis and Anticancer Pharmacology, Lebanese University, Hadath, Lebanon; 3 Department of Medical Laboratory Technology, Faculty of Applied Medical Sciences, King Abdulaziz University, Kingdom of Saudi Arabia; 4 Center of Excellence in Genomic Medicine Research, King Abdulaziz University, P.O. Box 80216 Jeddah 21589, Kingdom of Saudi Arabia; 5 KACST Technology Innovation Centre in Personalized Medicine, King Abdulaziz University, Jeddah, Saudi Arabia; 6 Zoology Department, Faculty of Science, Minia University, Minia, Egypt; 7 Department of Biochemistry, King Abdulaziz University, Kingdom of Saudi Arabia; 8 Dr. Rath Research Institute, Santa Clara, CA, USA; 9 Department of Biology, Faculty of Science, King Abdulaziz University, P.O. Box 80216 Jeddah 21589, Kingdom of Saudi Arabia; 10 Department of Animal and Veterinary Sciences, American University of Beirut, Lebanon; 11 King Abdulaziz University, Kingdom of Saudi Arabia

**Keywords:** Acute T-cell leukemia, Specific Nutrient Synergy, Human T-cell Lymphotropic Virus type I (HTLV-1), Tax, NF-κB pathway, MMP-9

## Abstract

**Background:**

Adult T-cell Leukemia (ATL) is a disease with no known cure. The disease manifests itself as an aggressive proliferation of CD4^+^ cells with the human T-cell Lymphotropic virus type 1 (HTLV-1). The leukemogenesis of the virus is mainly attributed to the viral oncoprotein. Tax activates the Nuclear Factor kappa B (NF-κB) which stimulates the activity and expression of the matrix metalloproteinase-9 (MMP-9). The objective of this study was to investigate the efficacy of a specific nutrient synergy (SNS) on proliferation, Tax expression, NF-κB levels as well as on MMP-9 activity and expression both at the transcriptional and translational levels in two HTLV-1 positive cell lines, HuT-102 and C91-PL at 48h and 96h of incubation. Cytotoxicity of Epigallocatechin-3-gallate (EGCG) was assayed using CytoTox 96 Non-radioactive and proliferation was measured using Cell Titer96^TM^ Nonradioactive Cell Proliferation kit (MTT- based assay). Enzyme linked immunosorbant assay (ELISA) and electrophoretic mobility shift assay (EMSA) were used to assess the effect of SNS on NF-**κ**B mobility. Zymography was used to determine the effects of SNS on the activity and secretion of MMP-9. The expression of MMP-9 was done using RT-PCR at the translational level and Immunoblotting at the transcriptional level.

**Results:**

A significant inhibition of proliferation was seen in both cell lines starting at a concentration of 200μg/ml and in a dose dependent manner. SNS induced a dose dependent decrease in Tax expression, which was paralleled by a down-regulation of the nuclearization of NF-κB. This culminated in the inhibition of the activity of MMP-9 and their expression both at the transcriptional and translational levels.

**Conclusions:**

The results of this study indicate that a specific nutrient synergy targeted multiple levels pertinent to the progression of ATL. Its activity was mediated through the NF-**κ**B pathway, and hence has the potential to be integrated in the treatment of this disease as a natural potent anticancer agent.

## Background

The human T-cell lymphotropic virus type 1 (HTLV1) is a retrovirus that infects CD4-positive T-cells resulting in the development of adult T-cell leukemia (ATL) in approximately 5% of the cases. ATL manifests as a consequence of the clonal expansion of mature and activated infected CD-4 positive T-cells and is associated with a poor prognosis due to its immunosuppressive and chemotherapy-resistant nature [1]. The causative agent of this disease, HTLV1, is transmitted via breast feeding, transplacentally from mother to child, through sexual contact and blood transfusion [2]; while in vivo transmission occurs through cell-to-cell contact [3]. The HTLV1 oncoprotein, Tax, has been shown to be vital for viral persistence and leukemogenesis due to its pleitoropic effects on cellular proliferation and apoptosis as well as viral replication [4]. As such, targeting Tax has become a novel approach in the treatment of ATL; however, the current options either failed to nullify the issue of relapse [5]. Therefore, it is essential to unravel a nontoxic compound having an inherent ability to inhibit Tax expression and a potential to serve as a therapeutic agent against this aggressive malignancy.

The successful documentation of the safety and effectiveness of botanical and dietary natural compounds in cancer prevention has led to the development of a nutrient mixture composed of ascorbic acid (AA), lysine, proline, arginine, epigallocatechingallate (EGCG) and other micronutrients [6]. This natural assortment of nutrients, also known as SNS has exhibited synergistic anticancer properties in a large number of solid cancer cell lines, blocking tumor growth, tumor invasion and MMP expression, both in vitro and in vivo [7]. Not only that, but SNS had an anti-proliferative effect against HTVL-1 positive and negative malignant T-lymphocytes and demonstrated pro-apoptotic effects with respect to HTLV-1 leukemic cells in specific, via the up-regulation of the pro-apoptotic proteins p53, p21 and Bax, and the down-regulation of the pro-survival Bcl2-α protein [8]. We have also reported enhanced antiproliferative activity of SNS in the presence of polyethylene glycol gold plated nano-particles [9].

SNS was in fact formulated based on the capacity of the individual components to alter key physiological pathways involved in cancer progression and metastasis [10]. In fact, ingredients of SNS were reported to inhibit the destruction of the extracellular matrix (ECM), which is a pre-requisite for cancer cell invasion and metastasis [11]. For example, the biosynthesis of collagen depends on an adequate supply of AA, the amino acids lysine and proline as well as the micronutrients manganese and copper [12]. Therefore, the integration of these nutrients into the formulation would result in strengthening the ECM. Not only that, but lysine is also a natural inhibitor of plasmin-induced proteolysis and, therefore, increases ECM stability by inhibiting the breakdown of collagen fibers [11]. Similarly, N-acetyl cysteine, AA, selenium and EGCG inhibited the invasiveness of tumor cells by blocking the activity of MMPs, which are a unique family of more than 20 proteases responsible for the proteolytic degradation of the ECM, which is essential for the dissemination of cancer cells to secondary sites [12]. The over-expression of MMPs, which is a common occurrence in malignant tumors, is correlated with tumor aggressiveness, stage and prognosis [11,12].

Additionally, multiple studies have associated the makeup of SNS with inhibitory activity against the transcription factor NF- κB, which is constitutively activated by HTLV-1 Tax protein and has as a critical role in the pathophysiology of ATL [13]. In fact, the inhibitory effect of EGCG on the activity of NF-kB has recently been demonstrated in ATL cell lines [14]. With respect to AA, it was able to dose-dependently repress activation of NFκB [Harakeh et al., unpublished data]. Furthermore, N-acetyl-cysteine (NAC) was found to reverse NF-κB binding to DNA and NF-κB-dependent oncogene expression in HaCaT cells [15]; while selenium compounds were capable of preventing esophageal carcinogenesis by inhibiting NF-kB activation [16]. Finally, magnesium deficiency induced NFκB expression in endothelial [17] and macrophage cells [18]. Nonetheless, the synergistic effect of the nutrient mixture on NF-kB activity in HTLV1 infected cells is yet to be determined.

The combination of these micronutrients is accompanied by the advantage of using lower doses of the separate constituents and has the potential of increasing the biological effect by establishing novel metabolic targets [6]. Therefore the objective of this study is to investigate the efficacy of SNS on Tax expression, NF-**κ**B levels as well as on MMP-9 activity and expression both at the transcriptional and translational levels in two HTLV-1 positive cell lines, HuT-102 and C91-PL thus determining the potential of using SNS in conjunction to ongoing treatment.

## Results and discussion

### Effect of non-cytotoxic concentrations of SNS on proliferation

To identify the doses to be used in subsequent experiments, we determined the range of non-cytotoxic concentrations of SNS against the C91-PL and HuT-102 HTLV1- positive cell lines used. Cells were grown in the presence of various concentrations of SNS ranging from 0 to 1000μg/ml. Concerning the effects of such doses on freshly activated human mononuclear lymphocytes, it was previously shown that the concentrations used in this study had no pronounced toxicity [8].

**Figure 1:**
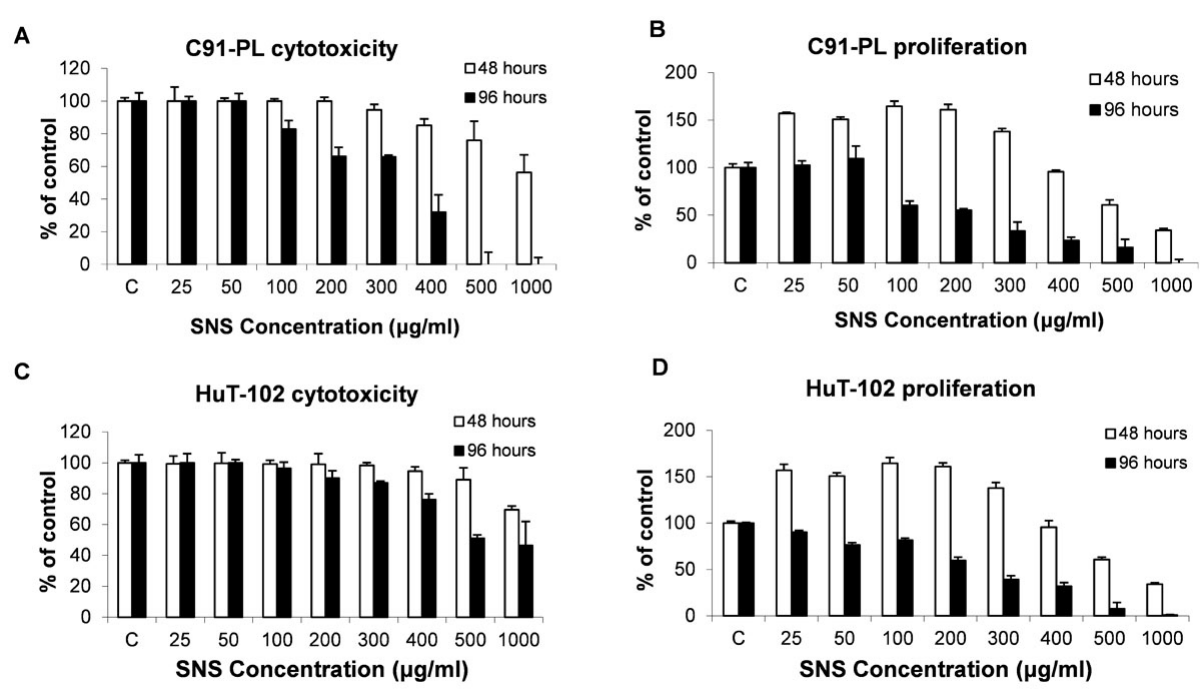
Cytotoxicity and anti-proliferative effects of SNS. Effect of SNS on cytotoxicity (A, C) and proliferation (B, D) of C91-PL and HuT-102 HTLV-1 positive cell lines respectively. Each value is the mean ± SD of three separate experiments done in triplicates.

There was a dose-dependent decline in the viable cell counts in the case of both two cell lines tested. The dose which resulted in the death of 50% of the cells was recorded (D_50_) (Figure 1A, C). The corresponding reduction in proliferation at D_50_ was also documented for the two cell lines (Figure 1B, D). For C91-PL cell line, the D_50_ was 772 and 365µg/ml at 48h and 96h of exposure respectively (Figure 1B) and was associated to a high anti-proliferative effect (*p* < 0.05) of 82% and 65% after 48h versus 96h in comparison to the control (Figure 1A). Similarly, the D_50_ HuT-102 cell line were respectively 586 and 500 µg/ml at 48h and 96h. A significant decrease in proliferation activity at these two aforementioned culture periods was respectively of 62% and 91% when compared to the control (Figure 1B).

The potent inhibitory concentrations of proliferation (<500μg/ml) found in this study were within the previously reported range of concentrations [9]. Moreover, while studies using solid cancer cell lines indicated an anti-proliferative effect in the first 24 hrs of treatment [6], this effect was observed in our cell lines starting at 48h to become more pronounced at 96h of treatment in the two cell lines (Figure 1). In fact, upon treating the cell lines with tested concentrations for 24h, there were no observed effects in either one of the two cell lines (Data not shown). The same lag was also observed upon treating the cells with EGCG [14,19] and AA [unpublished results, [20]]. This might indicate a requirement of the cells to undergo a few cycles of replications in the presence of the test compound for it to start exerting an effect.

Therefore in all the experiments that followed, where the incubation period was increased to 96h, only the non-cytotoxic concentrations of SNS (200-350 μg/ml) were applied to the two cell lines. Those concentrations were non-cytotoxic in primary cultures of freshly isolated normal human T-lymphocytes (Data not shown).

### Effect of SNS on Tax expression

Tax is 40 kDa phosphoprotein encoded by the pX region of the HTLV1 genome*.* It is a viral protein post-translationally modified by ubiquitination, phosphorylation and acetylation, which enable this oncoprotein to affect a plethora of cellular processes that work together to promote the survival and proliferation of infected cells [3]. The effect of SNS on viral proteins in general has not been investigated; however EGCG has been documented to suppress HTLV-I pX mRNA [21]*;* while treating HPV transfected HeLa cells with vitamin C down-regulated the protein expression of the viral oncoprotein E6 [22]. Moreover, these two major constituents of SNS induced a dose dependent decrease in Tax expression [[14], unpublished results].

**Figure 2:**
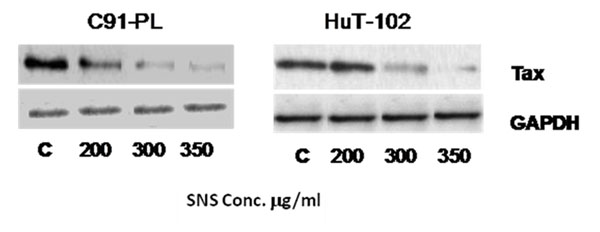
Effects of SNS on Tax expression in C91-PL and HuT-102 cell lines. GAPDH was used as a control. The immunoblots represent results obtained in three independent replicates.

The effect of SNS on the viral oncoprotein, Tax expression was studied by western blotting and GAPDH was used to ensure equal protein loading (Figure 2). The results revealed that SNS induced a dose dependent decrease on Tax translational expression levels in both HTLV-I positive cell lines (Figure 2). For the same concentration of 200μg/ml SNS showed more potency in reducing Tax protein levels in the C91-PL cell line compared to the HuT-102 cell line. However in both cell lines Tax expression was almost completely lost at a concentration of 350μg/ml.

### Effects of SNS on NF-κB activity

Tax has been shown to immortalize cells *in vitro* and to promote tumor formation in transgenic mice [3]. This is correlated with its intrinsic ability to alter the expression of a number of cellular genes by acting on multiple signaling pathways, the most prominent of which is NF-κB. The transcriptional factor NF-κB is rendered constitutively active by Tax in HTLV1-infected cells [3]. The constitutive activation of NF-κB by Tax due to the Tax’s ability to override this inhibitory mechanism by activating inhibitor of nuclear factor kappa-B kinase (IKK) or inducing the proteosomal degradation of the inhibitor of nuclear factor kappa-B (IkB). Moreover Tax acted in the nucleus either directly or indirectly to promote the interaction of the p65 subunit with coactivators that are necessary for its transcriptional activity [13].

To establish the effect of SNS on NF-κB activity, we used EMSA to identify the dimer combination of this transcription factor present in the nucleus. The results revealed that both HuT-102 and C91-PL harbored the p65/p50 heterodimer in their nuclei (data not shown).

**Figure 3:**
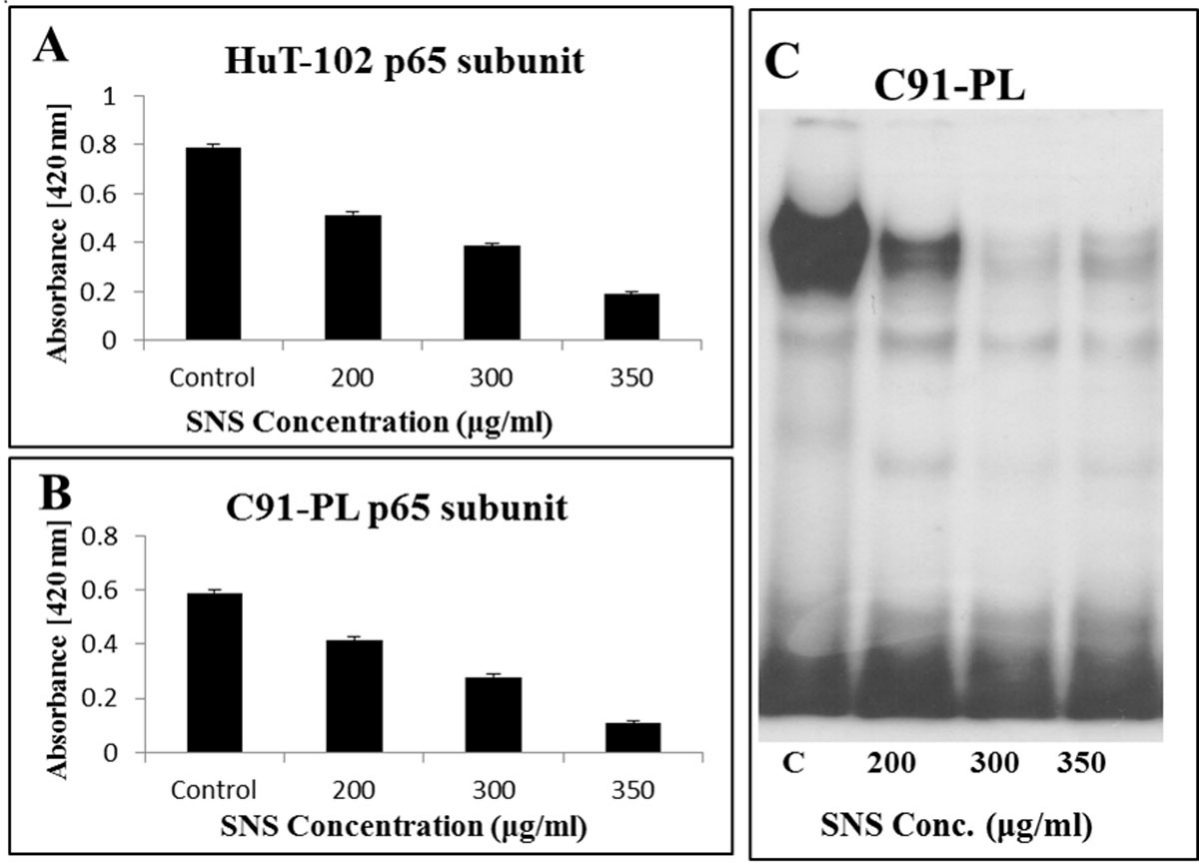
Effects of SNS on NF-κB activity and nuclear translocation in HuT-102 and C91-PL HTLV-1 positive cell lines. (A, B) Nuclear quantity of NF-κB assessed by ELISA done in triplicate. (C) EMSA gel is a representative of three independent experiments with nuclear C91-PL extract.

Nuclear extracts obtained from both cell lines were then subjected to ELISA, in order to quantitatively evaluate the impact of SNS on the relocation one of the heterodimer proteins p65 from the cytoplasm to the nucleus. The results showed that the test compound induced a dose dependent decrease in the nuclear levels of p65; where the inhibition was greater in C91-PL cells, reaching around 82%, and to a lesser extent in HuT-102 cells, reaching 75%. These reductions were obtained at the highest applied concentrations of SNS in both cell lines (Figure 3A, B).

It appears that SNS concurrently induced a dose dependent decrease in the binding of the p65/p50 heterodimer to its DNA consensus sequence in the C91-PL cell line (Figure 3C); in addition to the HuT-102 cell line (Data not shown). The binding specificity of p65 to NF-κB consensus probe was determined using mutant and cold probes (Data not shown).

The effects of the test compound on NF-κB may be indirect and due to its inhibitory effect on Tax which would be expected to inhibit the Tax induced constitutive activation of NF-κB by reversing the aforementioned mechanisms of action. Even though, there has not been any study that we know of linking SNS with NF-kB, there is overwhelming evidence correlating a number of its constituents with the expression and activity of this transcription factor. In fact EGCG, vitamin C and selenium inhibited the activation of NF-κB by blocking the activity of IKK [23,24]. Moreover, EGCG suppressed the activity of NF-κB in HeLa cervical cancer cells through the inhibition of phosphorylation and consequent degradation of IκBα and IκBβ [25]. Similarly AA inhibited the degradation of IκBα in human acute myeloid leukemia HL-60 cell lines [26]. Another constituent, NAC, suppressed the expression of NF-kB mediated proliferation genes, such as c-FLIP, Cyclin D and Bcl2 through the inhibition of IKK activity [27]. Therefore the inhibitory effect induced by SNS on NF-κB activity might be occurring indirectly, as a consequence of SNS’s effect on Tax expression or it might be due to the direct synergistic effect of the its various components on regulatory elements involved in the activation of this transcription factor.

### Effect of SNS on MMP-9 expression at the transcriptional level

MMPs are extracellular proteases that have been shown to favor cancer invasion and metastasis by ameliorating the infiltration of tumor cells from the primary site to secondary organs. The gelatinase MMP-9 has been especially correlated with the infiltration and metastasis of leukemia cells; where it has been reported, both in vitro and in vivo, that high MMP-9 expression levels are associated with clinically aggressive tumors and worse prognosis of non-Hodgkin's lymphomas (NHL) [28]. Moreover, it has been suggested that the invasiveness of ATL can be at least in part attributed to the over-expression of MMP9 in HTLV-1 positive cells [29].

**Figure 4:**
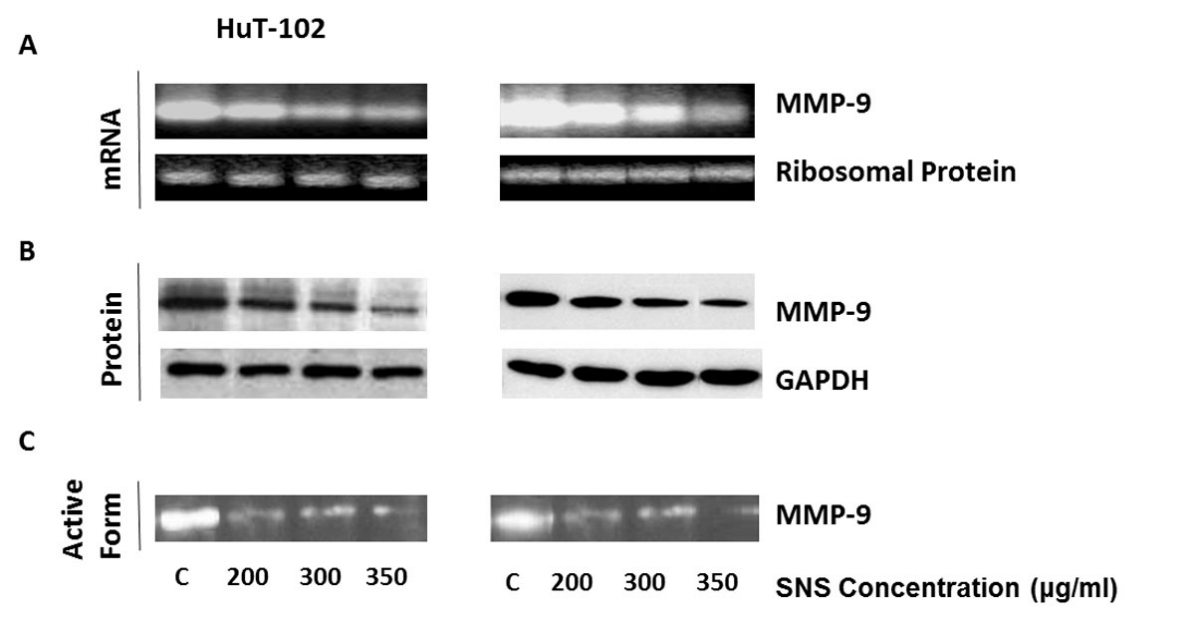
**Effects of SNS on MMP-9 expression and activity.** Effect of SNS on MMP-9 mRNA (A), protein (B) and activity (C) in two ATL-HTLV-1 positive cell lines. Equal loading was ensured using ribosomal protein for mRNA expression (A) and GAPDH for protein expression (B).The experiments were done in triplicates.

To investigate the effect of SNS on the transcriptional level of MMP-9, mRNA isolated from C91-PL and HuT-102 cells treated with non-cytotoxic concentrations of the test compound were exposed to RT-PCR which amplified and quantified their respective MMP-9 mRNA levels. The PCR products were then subjected to agarose gel electrophoresis. The results indicated that SNS induced a dose dependent decrease in MMP-9 mRNA levels in both cell lines and that this inhibitory effect started at a concentration of 200μg/ml of SNS (Figure 4A). Note that ribosomal phosphorprotein was used to ensure equal loading.

The results indicated that SNS down-regulated the mRNA expression levels of MMP-9 in a dose dependent manner starting at a concentration of 200μg/ml. The MMP-9 promoter harbors several putative NF-κB consensus sequences making this protease a target gene for the NF-κB transcription factor [29]. Accordingly, it is hypothesized that the SNS-induced reduction of MMP-9 transcription in the HTLV-1 infected cells was at least in part due to its inhibition of the NF-κB nuclear translocation and activity. In fact, a major constituent of SNS, EGCG, inhibited MMP-9 expression at the transcriptional level through the inactivation of NF-κB via the inhibition of PI3K/AKT activation in T24 human bladder cancer cells [30]. Similarly, it hampered MMP-2 and MMP-9 transcription in human prostate carcinoma DU-145 cells by inhibiting the activation of C-Jun and NF-κB transcription factors and the phosphorylation of p38 and ERK1/2 pathways [31]. Moreover, EGCG inhibited the EGF induced DNA binding activity of NF-κB to the MMP9 promoter and down-regulated the resulting expression and gelatinolytic activity of MMP9 in MDA-MB-231 human breast cancer cell line [32], while it modulated MMP9 expression by preventing the nuclear delocalization of NF-κB in lung carcinoma 95-D cells [33]. Moreover, EGCG was also reported to inhibit the EGF induced DNA binding activity of NF-κB to the MMP9 promoter and to down-regulate the resulting expression and gelatinolytic activity of MMP9 in MDA-MB-231 human breast cancer cell line [32]. In lung carcinoma 95-D cells, EGCG modulated MMP9 expression by preventing the nuclear delocalization of NF-κB [33]

### Effect of SNS on MMP-9 expression at the translational level

In general, there is no correlation between mRNA levels and their subsequent protein levels. In fact MMP-9 is known as a mRNA that having a highly structured 5’UTR rendering its translation dependent on the activity of the eukaryotic initiation factor 4E (eIF-4E). The latter being the rate-limiting and mRNA cap binding component of the eukaryotic initiation factor 4F (eIF-4F) translation initiation complex*.* In general, there is no correlation between mRNA levels and their subsequent protein levels. In fact, MMP-9 is considered a weak mRNA having a highly structured 5’UTR rendering its translation dependent on the activity of the eIF-4E*.* Nonetheless, a number of SNS constituents decreased the protein expression levels of this gelatinase independently from their activity on mRNA level [[14], Harakeh S unpublished data].

To further inspect the effect of the test compound on the concentration needed to affect MMP-9 protein levels, western blotting was used. Cells were incubated with various concentrations of SNS for 96h. The protein levels of MMP-9 were subsequently determined using specific antibodies. SNS clearly induced a dose dependent decrease in the translational levels of MMP-9 in both cell lines starting at a concentration of 200μg/ml (Figure 4B). Therefore, the translational levels of MMP9 seem to be equal to the transcriptional levels of its mRNA in their susceptibility to SNS (Figure 4A). To ensure equal protein loading, the GADPH was used.

### Effect of SNS on MMP-9 activity

MMPs are synthesized as inactive pro-enzymes [34] and their proteolytic activity is regulated by a well-organized pathway, which includes the conversion of plasminogen to plasmin that is crucial for MMP activation [9]. To evaluate the effect of SNS on MMP9’s activity, which correlates with the invasion potential of the two cell lines, zymography was used. As shown in Figure 4C, the test compound induced a dose dependent decrease in the activity of MMP-9 in both cell lines, also starting at 200μg/ml. This decrease seems to culminate in an almost complete inhibition of MMP-9’s gelatine degrading potential at 350μg/ml; which was especially obvious in the C91-PL cell line (Figure 4C).

In fact the lysine present in SNS is a natural inhibitor of plasmin-induced proteolysis and therefore might interfere with the activity of MMP-9 at this level. Moreover, lysine [9], AA (unpublished) and EGCG [14] inhibited MMP9 activity in a dose dependent manner in HTLV1 infected malignant T-cells. In addition, L-arginine decreased the activity of MMP-2 and MMP-9 in mdx muscle fibers [35] and in an isolated lung perfusion rat model of acute pulmonary embolism (APE) [36]. In addition, the supplementation of cord plasma and HUVEC line with physiological doses of magnesium sulfate led to the attenuation of MMP-9 activity [37]. Therefore, the SNS induced decrease in MMP-9 activity is in accordance with the previously published effect of its separate constituents.

## Conclusions

The results of this study showed for the first time that SNS induces a dose dependent inhibition of Tax expression, NF-κB activity as well as MMP9 activity and expression both at the transcriptional and translational levels starting at a concentration of 200μM in the two HTLV1 infected cell lines. Therefore, it seems that SNS targets multiple levels pertinent to the progression of ATL and hence has the potential to be integrated in the treatment of this disease as a natural, yet potent anticancer agent.

## Materials and methods

### SNS composition

1mg/ml of SNS solution contains 900 µM of ascorbate, 1.1 mM of lysine, 1.1 mM of proline, 500 µM of arginine, 250 µM of N-acetylcysteine, 150 µM of EGCG, 85 µM of Selenium, 7 µM of copper and 4 µM of manganese and 4 µM Calcium. Each SNS, whose source was reported earlier [8], was dissolved in RPMI 1640 media in stock solutions of 33.3 mg/ml, filter-sterilized using a 0.22µm filter, aliquoted and stored at -20°C until the day of the experiment. Each stored aliquot was used for just one experiment only and the left over was discarded.

### Cell lines

Two HTLV-1 positive ATL cell lines were used, namely HuT-102 and C91-PL (provided by Dr A. Gessain, Institut Pasteur Paris, France). The cells were grown in RPMI 1640 complete growth media with 25 mM of Hepes, supplemented with 10% Fetal Bovine Serum, 100 µg/ml of Streptomycin and 100 U/ml of Penicillin. Cells were routinely grown at 37°C in a 5% CO_2_-humidified incubator.

### Cell growth and cytotoxicity

Cytotoxicity of SNS at different concentrations was assayed using CytoTox 96 Non-radioactive Cytotoxicity Assay (Promega, Corp., Madison, WI) and proliferation was measured using Cell Titer96^TM^ Nonradioactive Cell Proliferation kit (Promega Corp., Madison, WI). Experiments were carried out according to the instructions of the manufacturer and as prescribed previously [38,39].

### ELISA and EMSA for NF-κB

The HTLV-1 positive cell lines were grown in the presence or absence of the test compound and harvested at the end of the experiment and nuclear extracts were obtained as previously described [14]. Protein concentrations were determined using the Bio-Rad DC Protein Assay Kit (BioRad Laboratories, Hercules, CA), with inclusion of bovine serum albumin as a standard. For the ELISA, the 96-well plate, supplied with the kit (Roche, Mannheim, Germany), was coated with anti-p65 antibody (Santa Cruz Biotechnology Inc., Santa Cruz, CA), and the procedure was followed according to the manufacturer’s instructions. For EMSA, NF-κB consensus oligonucleotides and mutant sequences (see Table 1) were end-labeled with γ-^32^P ATP, using T_4_-polynucleotide kinase, and the assay was performed as described previously [14].

**Table 1:** Oligonucleotides’ sequences and experimental conditions used for RT-PCR and EMSA analysis.

Experiment	molecule	Size (bp)	Sequence	# of Cycles	Hybridization Temp (°C)
**RT-PCR**	MMP-9	409	sense: 5’ CGCAGACATCGTCATCCAGT 3’ antisense: 5’ GGATTGGCCTTGGAAGATGA 3’	**30**	**62**
	
	Ribosomal phosphoprotein	486	sense: 5’ GTTCACCAAGGAGGACCTCA 3’ antisense: 5’ CACATTAGGCAGAGGTGTCT 3’	**25**	**50**

**EMSA**	NF-κB probe	22	sense: AGTTGAGGGGACTTTCCCAGGC antisense: GCCTGGGAAAGTCCCCTCAACT	-	**37**
	
	Mutant probe	22	sense: AGTTGAGGCGACTTTCCCAGGC antisense: GCCTGGGAAAGTCGCCTCAACT	-	**37**

### RT- PCR for MMP-9 mRNA expression

Both cells treated or untreated for 96h with the test compound were collected and stored at -80°C. Total RNA was extracted from the cells using NucleoSpin RNA II kit (Macherey, Nagel). After testing different RNA concentrations and assessed their quality control, two micrograms of mRNA were reverse transcribed into first strand cDNA using One Step RT-PCR kit (Ready Mix Version) (Abgene, Promega). Reactions was conducted in 50µl volume using specific oligonucleotide primers designed to detect MMP-9 and ribosomal phosphoprotein according to conditions shown in Table 1. Ribosomal phosphoprotein (NCBI: NM_022402.2) was used to ensure equal loading.

### Western blotting for MMP-9 and Tax proteins expression

The HTLV-1-positive cells treated or not with test compound were lysed in a buffer containing 50mM Tris–HCl, pH 7.5, 150mM NaCl, 1% Nonidet P40, 0.5% sodium deoxycholate, 4% protease inhibitors, and 1% phosphatase inhibitors. Western blotting was conducted as prescribed previously [14]. The Primary antibodies specific to MMP-9, Tax, and GAPDH were obtained from Santa Cruz Biotechnology Inc. (Santa Cruz, CA) and horseradish peroxidase-conjugated secondary antibody were purchased from (Bio-Rad, Hercules,CA). The detection procedure of the protein bands was performed using an enhanced chemiluminescence system, and banded proteins were developed on X-Ray film using a Xomat machine (Amersham, Pharmacia, Biotech).

### Zymography for MMP-9 activity

Cells were treated with various concentrations of SNS for three days and then starved, by removing the FBS from the growth medium, for 24 hours and treating the cells with various concentrations of the test compound. The zymography experiments were performed as previously described [14].

## Statistical analysis

The one-way analysis of variance (ANOVA) was used in data analysis. The discrepancies between the means of both treated and untreated control groups were tested for significance using Fisher’s least significant differences at *p*≤0.05 (Fisher PLSD). An effect was considered significant when the value (±) of mean difference between groups exceeded Fisher PLSD in the one factor ANOVA test.

## List of abbreviations

APE: Acute Pulmonary Embolism; ATL: Adult T-cell Leukemia; AA: Ascorbic Acid; EMSA: Electrophoretic Mobility Shift Assay; ELISA: Enzyme Linked Immunosorbant Assay; EGCG: Epigallocatechin-3-gallate; eIF-4F: Eukaryotic Initiation Factor 4F; ECM: Extracellular Matrix; HTLV-1: Human T-cell Lymphotropic Virus type 1; MMP-9: Matrix Metalloproteinase-9; NAC: N-Acetyl-Cysteine; NHL: Non-Hodgkin's Lymphomas; NF-κB: Nuclear Factor kappa B; SNS: Specific Nutrient Synergy

## Competing interest

All authors declare no competing interests

## Authors’ contributions

SH designed and supervised the study. AN and MR involved in study design. RA, EB and MDA performed the experiments and executed the results. SH, RA, MA and MAE contributed in data analysis and manuscript drafting and editing. EA, GAD and TK participated in data analysis and critically revised the manuscript. All authors have read and approved the final version of the manuscript.

## Declarations

Publication charges for this article have been funded by the Center of Excellence in Genomic Medicine Research (CEGMR), King Abdulaziz University, Jeddah, 21589, Kingdom of Saudi Arabia.

This article has been published as part of *BMC Cancer* Volume 15 Supplement 1, 2015: Selected articles from the 2nd International Genomic Medical Conference (IGMC 2013): Cancer. The full contents of the supplement are available online at http://www.biomedcentral.com/bmccancer/supplements/15/S1
